# Associations Between Circulating Spexin, Obesity, and Insulin Resistance in Korean Children and Adolescents

**DOI:** 10.3390/nu17193177

**Published:** 2025-10-08

**Authors:** Shin-Hee Kim, Yoon Hong Chun

**Affiliations:** Department of Pediatrics, Incheon St. Mary’s Hospital, College of Medicine, The Catholic University of Korea, Seoul 06591, Republic of Korea; kshped@catholic.ac.kr

**Keywords:** spexin, obesity, insulin resistance, children, adolescents

## Abstract

**Background:** Spexin is a neuropeptide involved in various physiological functions, including energy metabolism, appetite regulation, and weight loss. This study aimed to identify correlations between circulating spexin levels, obesity, and insulin resistance (IR) in Korean children and adolescents. **Methods:** We included 128 Korean children and adolescents in the study. Among them, 69 individuals (53.9%) were classified as obese, 43 (33.6%) were considered overweight, and 16 (12.5%) had a normal weight. We recorded participants’ anthropometric parameters, fasting biochemical parameters, and homeostasis model assessment for insulin resistance (HOMA-IR), and assessed their correlations with plasma spexin levels. **Results:** Plasma spexin levels were significantly lower in obese subjects than in controls (mean, 163.1 vs. 198.4 pg/mL; *p* = 0.01). Subjects with IR had lower spexin levels than those without IR (mean, 145.3 vs. 185.1 pg/mL; *p* < 0.001). Spexin levels were negatively correlated with the BMI SDS (r = −0.30; *p* < 0.001), systolic blood pressure (r = −0.33; *p* < 0.001), fasting insulin (r = −0.41; *p* < 0.001), HOMA-IR value (r = −0.41; *p* < 0.001), triglyceride (TG) level (r = −0.38; *p* < 0.001), and plasma leptin level (r = −0.26; *p* = 0.004). In multivariate analysis, HOMA-IR and TG levels were independently associated with plasma spexin levels (*p* < 0.001 for both). Mediation analyses suggest a potential bidirectional relationship between obesity-related reductions in circulating spexin and insulin resistance. **Conclusions:** Decreased circulating spexin levels were associated with obesity and IR among Korean children and adolescents. Our findings suggest a link between circulating spexin, obesity, and IR in this population.

## 1. Introduction

Obesity in children and adolescents is of particular concern because it increases the risk of cardiometabolic diseases—such as hypertension, type 2 diabetes mellitus, metabolic syndrome, and nonalcoholic fatty liver disease—both in childhood and later in life [1,2]. Moreover, childhood obesity is linked to psychosocial complications, which can have lasting effects on mental health and well-being [3]. The ongoing health issues associated with childhood obesity place a heavy burden on the community.

Excessive accumulation of adipose tissue is a hallmark of obesity. As an endocrine organ, adipose tissue secretes adipokines that are critical for metabolic homeostasis [4,5]. Spexin is a 14–amino acid peptide hormone that was first identified using a bioinformatics approach in 2017 [6]. Spexin is secreted from adipose tissue, stomach, liver, and pancreas [7,8,9,10,11]. Its cognate receptors, galanin receptor 2 (GALR2) and galanin receptor 3 (GALR3), are expressed in both the CNS and peripheral tissues [12,13,14]. This neuropeptide acts as a systemic anorexigenic signal to the hypothalamus, reducing food intake and potentially increasing energy expenditure. In peripheral tissues, spexin promotes lipolysis, suppresses lipogenesis and hepatic fat accumulation, and alleviates insulin resistance (IR) and chronic inflammation in metabolic tissues [15]. Through its central and peripheral actions, spexin plays essential roles in feeding behavior, gastrointestinal motility, obesity, and diabetes [16,17]. Several studies on this hormone have focused on circulating spexin. Adipose tissue is considered to contribute to circulating spexin levels [18]. Decreased circulating spexin levels were observed in adult patients with obesity [18,19,20,21], diabetes mellitus [22], metabolic syndrome [23], and non-alcoholic fatty liver disease [24]. Physical exercise in obese adults has been shown to increase plasma spexin levels and improve metabolic profiles, especially in individuals who respond positively to exercise interventions [25].

Limited studies exist on the association between circulating spexin, obesity, and IR in children [26,27,28]. Based on adult evidence, we hypothesized that circulating spexin levels in Korean children and adolescents are associated with obesity and obesity-related parameters. Moreover, it remains unclear whether decreased circulating spexin and IR, both associated with obesity, are causally upstream of each other or downstream consequences [15]. Here, we evaluated the associations between spexin levels, obesity, and IR in Korean children.

## 2. Materials and Methods

We screened all children and adolescents who attended our pediatric endocrinology clinic from February 2024 to January 2025. After excluding those with endocrine disorders, genetic disorders, or other chronic diseases, 174 were eligible for inclusion in the study. Thirty-three individuals declined to participate, and 13 who had consented but lacked available fasting blood samples were excluded from the final analysis. Finally, 128 participants were enrolled: 69 (53.9%) in the obese group, 43 (33.6%) in the overweight group, and 16 (12.5%) in the normal-weight group. Participants were categorized as having obesity, being overweight, or having a normal weight according to body mass index (BMI). Obesity was defined as a BMI at or above the 95th percentile for age and sex; overweight, as a BMI from the 85th to just below the 95th percentile; and normal weight, as a BMI from the 15th to just under the 85th percentile. The control group comprised individuals who presented with concerns about endocrine disorders (e.g., precocious puberty or short stature) but were determined to be within normal limits and were being regularly followed in the outpatient clinic to monitor growth and pubertal development. We obtained written informed consent from all participants and their parents. The protocol received approval from the Institutional Review Board of Incheon St. Mary’s Hospital (IRB No. OC24TISI0020).

### 2.1. Anthropometric and Laboratory Measurements

We measured anthropometric parameters in all participants and computed SDSs using Korean reference data stratified by age and sex [29]. Pediatric endocrinologists assessed the pubertal stage using the Marshall and Tanner criteria [30]. Venous blood samples for biochemical measurements were collected after an overnight fast. The homeostasis model assessment for insulin resistance (HOMA-IR) was calculated to evaluate IR, determined by the formula: fasting insulin (µU/mL) multiplied by fasting blood glucose (mg/dL) divided by 405. Insulin resistance was defined via HOMA-IR with age-stage thresholds of ≥2.5 for prepubertal individuals and ≥4.0 for pubertal individuals [31]. Plasma spexin and leptin levels were measured using commercial ELISA kits from MyBioSource (San Diego, CA, USA) and Phoenix Pharmaceuticals (Belmont, CA, USA), respectively.

### 2.2. Statistical Analysis

We presented categorical variables as the number of subjects (%) and continuous variables as mean ± SD. Group comparisons used chi-square or Fisher’s exact tests for categorical data and Student’s t-test (two groups) or one-way ANOVA (more than two groups) for continuous data. Associations between spexin and continuous variables were examined using Pearson’s and partial correlations. Independent associations between spexin and clinical parameters were evaluated with multiple linear regression, including covariates significant in simple linear regression. Two separate mediation analyses tested (i) whether spexin mediated the association between obesity and IR and (ii) whether IR mediated the association between obesity and spexin. When the direct effect is non-significant but the indirect (mediated) effect is significant, this indicates complete mediation. When both the direct and indirect effects are significant, the relationship is classified as partial mediation. [32,33]. All analyses were conducted in R 4.3.3 (R Foundation for Statistical Computing, Vienna, Austria). Statistical significance was set at *p* < 0.05.

## 3. Results

### 3.1. Clinical Characteristics of the Study Subjects

Table 1 details the study participants’ demographic, clinical, and laboratory profiles. Half of the subjects (*n* = 64; 50%) were male, and the mean age of all participants was 10.3 years. Insulin resistance was observed in 42.0% of the obese subjects and 16.3% of the overweight subjects, whereas it was not present in any participants in the normal-weight group.

### 3.2. Association Between Plasma Spexin with Categorical Variables

**Figure 1:**
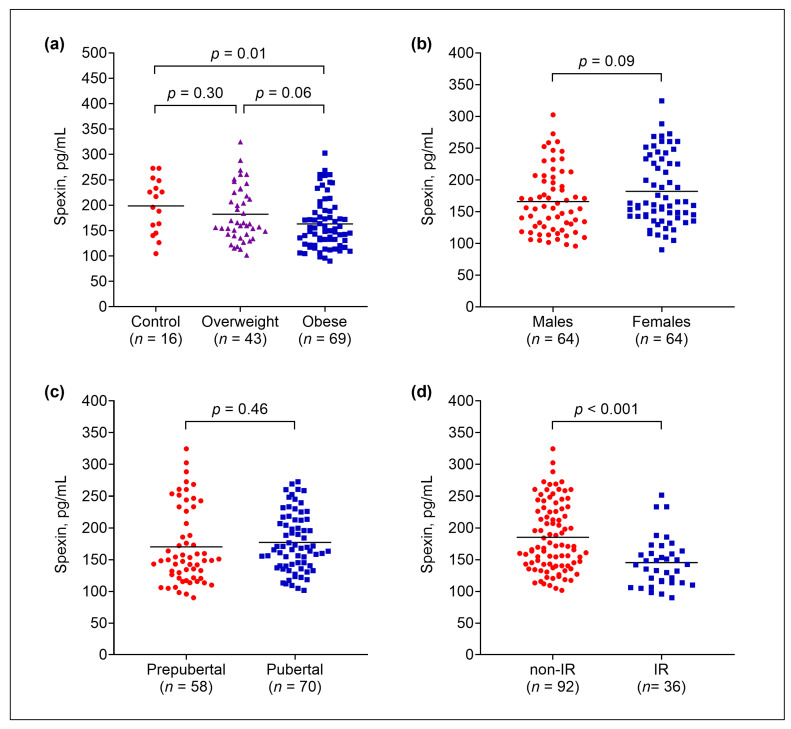
Circulating spexin levels by body weight (**a**), sex (**b**), pubertal stage (**c**), and insulin resistance status (**d**). Horizontal lines indicate mean values. **Abbreviations:** IR, insulin resistance.

Obese subjects had lower spexin levels compared to normal-weight subjects (mean, 163.1 pg/mL vs. 198.4 pg/mL; *p* = 0.01) (Figure 1a). There was no significant difference in spexin levels between males and females, with means of 165.9 pg/mL and 181.9 pg/mL, respectively (*p* = 0.09) (Figure 1b). Similarly, spexin levels did not differ between prepubertal and pubertal participants, with means of 170.0 pg/mL and 177.2 pg/mL, respectively (*p* = 0.46) (Figure 1c). Subjects with IR had lower spexin levels than those without IR (mean, 145.3 vs. 185.1 pg/mL; *p* < 0.001) (Figure 1d). This finding was consistent in both males (mean, 135.0 vs. 173.8 pg/mL; *p* = 0.01) and females (mean, 151.1 vs. 199.2 pg/mL; *p* < 0.001). In the obese group, spexin levels were also lower in the IR group compared to the non-IR group (mean, 139.0 pg/mL vs. 180.5 pg/mL; *p* < 0.001). No significant difference was observed in spexin levels between obese and normal-weight subjects without IR (mean, 180.5 pg/mL vs. 198.4 pg/mL; *p* = 0.26).

### 3.3. Correlations Between Plasma Spexin and Continuous Variables

Table 2 summarizes the simple correlations between plasma spexin and continuous variables. Plasma spexin was negatively correlated with BMI SDS (*r* = −0.30, *p* < 0.001), systolic blood pressure (BP) (*r* = −0.33, *p* < 0.001), fasting insulin (*r* = −0.41, *p* < 0.001), HOMA-IR (*r* = −0.41, *p* < 0.001), triglycerides (TG) (*r* = −0.38, *p* < 0.001), and plasma leptin (*r* = −0.26, *p* = 0.004). In analyses restricted to males, these associations persisted except for the spexin–BMI SDS association. In analyses restricted to females, these associations also persisted except for the spexin–leptin association. A negative association between spexin and total cholesterol was observed in females (*r* = −0.33, *p* = 0.009) but not in males (*r* = 0.05, *p* = 0.68). Among all participants, partial correlations controlling for age, sex, and BMI-SDS indicated that spexin remained significantly associated with systolic BP (*r* = 0.26, *p* = 0.004), fasting insulin (*r* = −0.30, *p* < 0.001), HOMA-IR (*r* = −0.32, *p* < 0.001), and TG (r = −0.40, *p* < 0.001). Furthermore, multiple linear regression analysis identified independent associations of spexin levels with HOMA-IR (*p* = 0.01) and TG values (*p* < 0.001) (Table 3).

### 3.4. Mediation Analysis

**Figure 2:**
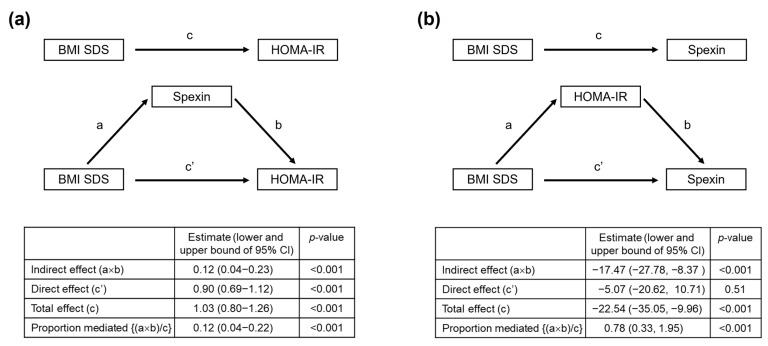
Mediation models for the relationship between obesity, insulin resistance, and spexin level. Two separate mediation analyses tested (**a**) whether spexin mediated the association between obesity and IR and (**b**) whether IR mediated the association between obesity and spexin.

Two separate mediation analyses were conducted to assess whether circulating spexin mediates the effect of obesity on IR (Figure 2a) and whether circulating spexin is a consequence of obesity-induced IR (Figure 2b). In the first mediation model, spexin served as a partial mediator linking BMI SDS to IR; the indirect (causal) effect via spexin accounted for 12% of the total effect (Figure 2a). In the second mediation model, IR served as a complete mediator of the effect of BMI SDS on spexin; the indirect (causal) effect via IR accounted for 88% of the total effect (Figure 2b). The findings suggest that obesity-related reductions in circulating spexin are largely a consequence of IR, whereas spexin plays only a limited, partial mediating role in the pathway from obesity to IR. The findings suggest that obesity-related reductions in circulating spexin are largely a consequence of IR, while spexin plays only a limited, partial mediating role in the pathway from obesity to IR.

## 4. Discussion

We evaluated the associations between spexin level, obesity, and IR in 128 Korean children and adolescents. Our research found that spexin levels were lower in the obese group compared to the control group and also lower in the IR group compared to the non-IR group. To the best of our knowledge, this study is the first to assess the association between spexin, obesity, and IR in Korean children and adolescents.

Our finding of decreased circulating spexin levels in obese children and adolescents is consistent with previous findings from studies conducted in adults [18,19,20,21] and in the pediatric population [26,27,28,34]. The observed decrease in spexin levels in obese individuals may result from decreased expression in fat tissue, which is an important source of spexin. In a prior study, spexin gene expression was down-regulated 15-fold in omental and subcutaneous fat in obese patients [18]. We found in our work that lower circulating spexin levels were present in the IR group compared to the non-IR group. This association has also been observed previously in children [26,28] and adult patients with non-alcoholic fatty liver disease [24]. In obese children who underwent Roux-en-Y gastric bypass, postoperative weight loss was associated with increased spexin levels, which were negatively correlated with HOMA-IR values [35]. The protective effect of spexin on IR has been evaluated in experimental studies [36,37,38]. Exogenous administration of spexin in high-fat diet–induced mice and rats decreased IR [36,37]. Spexin ameliorates obesity-induced IR by improving white adipose tissue browning via activation of the JAK2/STAT3 signaling pathway [38]. Although the relationship between decreased circulating spexin and IR remains unclear, two non-mutually exclusive explanations are plausible [15]. First, obesity-related adipose dysfunction drives chronic low-grade inflammation and dysregulates adipokine profiles (e.g., lower spexin and higher leptin), which are key mechanisms underlying obesity-induced IR [4,5,15,39]. Second, lower circulating anorexigenic spexin may reflect a compensatory downregulation intended to increase food intake in an insulin-resistant milieu (e.g., hyperinsulinemia), rather than acting as a primary causal driver [15]. In mice, glucose upregulated spexin mRNA expression in the glandular stomach. The glucose-induced increases in serum spexin and gastric (glandular stomach) spexin mRNA were blocked by insulin [10]. Our mediation analyses suggest a potential bidirectional relationship between obesity-related reductions in circulating spexin and IR. However, the results more strongly support decreased spexin as a downstream consequence of IR. Accordingly, obesity-induced IR could lower circulating spexin levels, which in turn may increase food intake and further aggravate IR, potentially creating a vicious cycle. Confirmation of this hypothesis will require additional longitudinal or functional studies.

In contrast to spexin, circulating leptin levels were higher in our obese children and inversely correlated with spexin levels, consistent with findings in both children [18,40] and adults [20]. Leptin is a satiety hormone that sends signals to the brain to modulate food intake and sustain energy homeostasis [41]. In this context, the inverse association between spexin and leptin may suggest a potential role for spexin in regulating body weight and energy homeostasis. Several studies have highlighted the importance of spexin in controlling obesity-induced leptin resistance by modulating leptin production in adipose tissue and leptin signaling in the brain [15]. Although the molecular and signaling interactions between spexin and leptin remain unclear, several studies suggest that spexin reduces leptin resistance, which appears to be related to improved LepRb-associated signaling, thereby enhancing the expression of LepRb and the melanocortin-4 receptor in the hypothalamus [15].

Spexin levels were inversely correlated with serum TG levels, consistent with previous reports [8,28], whereas other studies did not report this association [34,42]. Experimental studies using mice or rats fed a high-fat diet have also yielded inconsistent results [38,43]. One study found that TG levels were lower in the spexin-treated group compared to the control group [43], while another study showed that serum TG levels were comparable between the two groups [38]. Consistent with our findings, studies have reported that spexin levels are negatively correlated with BP, particularly in individuals with metabolic syndrome and obesity [25,44]. In rats with high-fructose diet–induced metabolic syndrome, spexin administration attenuates elevated BP, suggesting a potential therapeutic role [44]. In this study, simple linear regression analysis revealed a negative correlation between spexin and systolic BP, but this correlation disappeared in multiple linear regression. These discrepancies highlight the need for further research to clarify the specific conditions under which spexin influences parameters related to cardiovascular disease.

This study has several limitations. First, data were collected from a single-center cohort, which may limit the generalizability of the findings to other populations. Second, the number of normal-weight participants was relatively small compared with the overweight and obese groups, and this imbalance may have reduced statistical power. Thirdly, body composition, physical activity, diet, and pubertal hormones were not measured. While BMI was used as an obesity indicator, body composition, waist circumference, and skinfold thickness provide more reliable assessments of adiposity. Omitting these variables limits a mechanistic interpretation of the impact of spexin on IR. Lastly, we did not perform hyperinsulinemic–euglycemic clamp studies; therefore, IR was inferred from fasting surrogate indices, such as HOMA-IR, which may not fully capture clamp-defined insulin sensitivity. Reliance on these proxies, while practical, sacrifices the direct physiological insight of the clamp and may miss subtle changes in insulin sensitivity [45].

## 5. Conclusions

This study corroborates previously reported associations among spexin, obesity, and IR in Korean children and adolescents. Our mediation analyses further suggest a bidirectional interaction between obesity-related IR and reduced circulating spexin. Future studies should employ longitudinal and mechanistic designs to clarify bidirectionality, establish causality, and define the clinical utility of circulating spexin in obesity-related IR.

## Figures and Tables

**Table 1:** Clinical and laboratory data for obese, overweight, and normal-weight subjects.

Characteristic	Obese (*n* = 69)	Overweight (*n* = 43)	Control (*n* = 16)	*p* Value
Age, years	9.8 ± 2.4	10.6 ± 2.1	11.3 ± 2.3	0.04
Sex, no. (%)				
Male	45 (65.2)	15 (34.9)	4 (25.0)	0.001
Female	24 (34.8)	28 (65.1)	12 (75.0)	
Height SDS	1.1 ± 0.9	0.3 ± 0.8	0.2 ± 1.1	<0.001
Weight SDS	2.1 ± 0.5	1.0 ± 0.4	0.7 ± 0.7	<0.001
BMI, kg/m^2^	25.7 ± 2.2	21.7 ± 1.8	20.1 ± 1.3	<0.001
BMI SDS	2.4 ± 0.3	1.3 ± 0.2	0.7 ± 0.2	<0.001
Tanner stage				0.17
1	37 (53.6)	17 (39.5)	4 (25.0)	
2	15 (21.7)	14 (32.6)	4 (25.0)	
3	11 (16.0)	8 (18.6)	3 (18.8)	
4	2 (2.9)	1 (2.3)	3 (18.8)	
5	4 (5.8)	3 (7.0)	2 (12.5)	
Puberty, no. (%)	32 (46.4)	26 (60.5)	12 (75.0)	0.08
Systolic BP, mmHg	108.8 ± 9.4	105.1 ± 6.7	102.9 ± 3.5	0.009
Diastolic BP, mmHg	63.5 ± 6.3	62.8 ± 4.8	66.1 ± 8.8	0.20
HbA1c, %	5.4 ± 0.2	5.4 ± 0.2	5.4 ± 0.3	0.35
Glucose, mg/dL	92.8 ± 7.4	91.8 ± 8.7	90.4 ± 6.7	0.50
Insulin, μU/mL	13.7 ± 4.6	10.1 ± 3.4	5.5 ± 1.9	<0.001
HOMA-IR	3.1 ± 1.1	2.3 ± 0.8	1.2 ± 0.4	<0.001
Insulin resistance, no. (%)	29 (42.0)	7 (16.3)	0	<0.001
Total cholesterol, mg/dL	175.6 ± 23.0	159.6 ± 19.6	144.9 ± 11.1	<0.001
LDL cholesterol, mg/dL	114.9 ± 23.1	105.8 ± 17.3	98.8 ± 16.2	0.006
HDL cholesterol, mg/dL	49.9 ± 11.9	54.9 ± 9.4	57.3 ± 8.1	0.01
Triglycerides, mg/dL	119.3 ± 28.5	120.9 ± 20.6	113.8 ± 19.0	0.62
AST, U/L	23.1 ± 6.1	22.7 ± 4.5	21.2 ± 9.2	0.56
ALT, U/L	22.3 ± 8.4	18.6 ± 5.8	15.8 ± 4.6	0.001
ALP, U/L	267.8 ± 73.3	256.7 ± 70.4	251.4 ± 65.8	0.60
GGT, U/L	17.8 ± 8.2	14.2 ± 5.4	14.0 ± 5.8	0.02
Uric acid, mg/dL	5.1 ± 1.3	4.8 ± 1.0	4.6 ± 1.3	0.27
25-hydroxyvitamin D, pg/mL	14.5 ± 7.6	13.9 ± 6.8	15.1 ± 6.5	0.85
Spexin, pg/mL	163.1 ± 49.7	182.2 ± 53.7	198.4 ± 53.3	0.02
Leptin, pg/mL	17.5 ± 6.8	12.8 ± 6.9	13.7 ± 6.8	0.001

Data are presented as mean ± standard deviation values, unless otherwise indicated. **Abbreviations:** ALP, alkaline phosphatase; ALT, alanine transaminase; AST, aspartate transaminase; BMI SDS, body mass index standard deviation score; BP, blood pressure; GGT, gamma-glutamyl transferase; HbA1c, hemoglobin A1c; HDL, high-density lipoprotein; HOMA-IR, homeostasis model assessment-insulin resistance; LDL, low-density lipoprotein.

**Table 2:** Simple correlations of spexin levels with the clinical data.

Characteristic	All Subjects (*n* = 128)			Males (*n* = 64)			Females (*n* = 64)	
	*r*	*p*-Value		*r*	*p*-Value		*r*	*p*-Value
Age, years	0.03	0.76		0.05	0.72		0.14	0.25
BMI SDS	−0.30	<0.001		−0.12	0.34		−0.39	0.002
Systolic BP	−0.33	<0.001		−0.30	0.02		−0.33	0.008
Diastolic BP	−0.10	0.25		−0.17	0.18		−0.06	0.63
Tanner stage	0.01	0.90		0.06	0.65		0.06	0.63
HbA1c	−0.14	0.13		−0.19	0.13		−0.10	0.42
Glucose	−0.08	0.35		0.01	0.91		−0.18	0.16
Insulin	−0.41	<0.001		−0.35	0.004		−0.44	<0.001
HOMA-IR	−0.41	<0.001		−0.36	0.003		−0.44	<0.001
Total cholesterol	−0.16	0.07		0.05	0.68		−0.33	0.009
LDL cholesterol	−0.08	0.38		−0.02	0.89		−0.09	0.47
HDL cholesterol	0.11	0.20		0.04	0.75		0.21	0.09
Triglycerides	−0.38	<0.001		−0.42	<0.001		−0.43	<0.001
AST	0.15	0.09		0.11	0.38		0.19	0.13
ALT	−0.06	0.49		0.06	0.64		−0.14	0.27
ALP	−0.14	0.13		−0.24	0.06		−0.05	0.68
GGT	−0.11	0.23		−0.03	0.83		−0.13	0.31
Uric acid	−0.02	0.85		0.20	0.12		−0.22	0.08
25-hydroxyvitamin D	0.13	0.19		0.12	0.38		0.14	0.31
Leptin	−0.26	0.004		−0.28	0.03		−0.21	0.09

**Abbreviations:** ALP, alkaline phosphatase; ALT, alanine transaminase; AST, aspartate transaminase; BMI SDS, body mass index standard deviation score; BP, blood pressure; GGT, gamma-glutamyl transferase; HbA1c, hemoglobin A1c; HDL, high-density lipoprotein; HOMA-IR, homeostasis model assessment-insulin resistance; LDL, low-density lipoprotein.

**Table 3:** Simple and multiple linear regression analyses of variables associated with spexin levels.

	Simple Linear Regression					Multiple Linear Regression			
	Unstandardized Coefficients		Standardized Coefficients			Unstandardized Coefficients		Standardized Coefficients	
Variables	B	SE	β	*p*-Value		B	SE	β	*p*-Value
BMI SDS	−22.54	6.36	−0.30	<0.001		−4.04	7.50	−0.05	0.59
Systolic BP	−2.11	0.53	−0.33	<0.001		−1.05	0.53	−0.17	0.051
HOMA-IR	−18.98	3.71	−0.41	<0.001		−11.75	4.65	−0.26	0.01
Triglycerides	−0.81	0.17	−0.38	<0.001		−0.58	0.17	−0.28	<0.001

**Abbreviations:** BMI SDS, body mass index standard deviation score; BP, blood pressure; HOMA-IR, homeostasis model assessment-insulin resistance; SE, standard error.

## Data Availability

The datasets used and/or analyzed during the current study are available from the corresponding author upon reasonable request.

## Abbreviations

IR, insulin resistance; BMI SDS, body mass index standard deviation score; HOMA-IR, homeostasis model assessment of insulin resistance.
